# Brain-to-Brain Coupling in the Gamma-Band as a Marker of Shared Intentionality

**DOI:** 10.3389/fnhum.2020.00295

**Published:** 2020-07-30

**Authors:** Paulo Barraza, Alejandro Pérez, Eugenio Rodríguez

**Affiliations:** ^1^Center for Advanced Research in Education, University of Chile, Santiago, Chile; ^2^Institute of Education, University of Chile, Santiago, Chile; ^3^MRC Cognition and Brain Sciences Unit, University of Cambridge, Cambridge, United Kingdom; ^4^School of Psychology, Pontifical Catholic University of Chile, Santiago, Chile

**Keywords:** cooperation, hyperscanning, EEG, gamma, shared intentionality

## Abstract

Cooperation and competition are two ways of social interaction keys to life in society. Recent EEG-based hyperscanning studies reveal that cooperative and competitive interactions induce an increase in interbrain coupling. However, whether this interbrain coupling effect is just a reflection of inter-subject motor coordination or can also signal the type of social interaction is unknown. Here, we show that behavioral coordination and social interaction type can be distinguished according to the frequency of oscillation in which the brains are coupled. We use EEG to simultaneously measure the brain activity of pairs of subjects, while they were performing a visual cue-target task in a cooperative and competitive manner. Behavioral responses were quasi-simultaneous between subject pairs for both competitive and cooperative conditions, with faster average response times for the competitive condition. Concerning brain activity, we found increased interbrain coupling in theta band (3–7 Hz) during cooperation and competition, with stronger coupling during competitive interactions. This increase of interbrain theta coupling correlated with a decrease in reaction times of the dyads. Interestingly, we also found an increase in brain-to-brain coupling in gamma band (38–42 Hz) only during cooperative interactions. Unlike the theta coupling effect, the gamma interbrain coupling did not correlate with dyads’ reaction times. Taken together, these results suggest that theta interbrain coupling could be linked to motor coordination processes common to cooperative and competitive interactions, while gamma brain-to-brain coupling emerges as an electrophysiological marker of shared intentionality during cooperative interactions.

## Introduction

Cooperation and competition are two forms of social exchange that play a key role in social and cultural life (Tomasello, 2009). In functional terms, both types of social behavior involve mentalization processes (Gallagher et al., 2002; Elliott et al., 2006), but they differ markedly in terms of the intention that motivates the interaction (Tomasello and Carpenter, 2007; Tsoi et al., 2016). Specifically, the intention of participants during cooperative interactions is to obtain collective benefits over individual achievements, while during competitive interactions, the intention of participants is to maximize individual achievement at the expense of the other. Traditionally, neurocognitive commonalities and differences between both forms of social interactions have been studied in isolated subjects (Amodio and Frith, 2006; Lieberman, 2007). For instance, Decety et al. (2004) used fMRI to evaluate the hemodynamic activity of 12 adults while playing a cooperative or competitive game with a confederate. They found that cooperative behavior was selectively associated with orbitofrontal cortex activity, while competitive behavior was associated with inferior parietal and medial prefrontal regions. They also reported that both types of behavior activated a shared frontoparietal network related to executive functions. Although interesting, it is important to keep in mind that cooperation and competition are not distinctive attributes or properties of individual subjects. Both behaviors are best characterized as emergent processes arising in the dynamic coupling with others (Fantasia et al., 2014). Thus, to achieve a thorough understanding of the neural processes that underlie cooperative and competitive behaviors, it is crucial to elucidate how the brain activity of subjects engaged in social interactions is co-regulated and integrated to produce a flow of shared social experiences.

To evaluate the interbrain dynamics during social interactions, a change in the experimental approach is required. Recently, the “individualistic approach” in the study of social cognition has taken a “relational turn” (Schilbach et al., 2013), due to the use of hyperscanning setup consisting in simultaneously recording the brain activity of two or more people while interacting (Montague et al., 2002; Dumas et al., 2011; Czeszumski et al., 2020). To date, the results of hyperscanning studies –carried out from laboratory settings (Dumas et al., 2010; Pérez et al., 2017) to ecological contexts (Toppi et al., 2016; Dikker et al., 2017)– have been consistent in demonstrating that when people engage in social interactions, the activity of their brains is temporarily coupled, forming a brain-to-brain network (Hasson et al., 2012). For instance, NIRS-based hyperscanning revealed a differential co-activation of the prefrontal cortex between subjects during cooperative and competitive tasks (Cui et al., 2012; Cheng et al., 2015; Osaka et al., 2015; Balconi and Vanutelli, 2017a; Pan et al., 2017; Miller et al., 2019). In terms of the time-frequency domain, EEG-based hyperscanning shows the emergence of brain-to-brain oscillatory networks during social interactions (Lindenberger et al., 2009; Sänger et al., 2012; Astolfi et al., 2014; Hu et al., 2018; Pérez et al., 2019). In particular, recent reviews have shown that cooperative and competitive interactions would induce the transient formation of brain-to-brain couplings in theta (3–7 Hz) and alpha (8–12 Hz) bands (Balconi and Vanutelli, 2017b; Liu et al., 2018). It is important to note the fact that interbrain coupling at low frequencies has also been found in studies in which participants are asked to simply imitate movements between them (Tognoli et al., 2007; Dumas et al., 2010), casting doubts about whether brain-to-brain coupling at low-frequency informs us of complex cognitive processes linked to social interactions or is only an epiphenomenon derived from performing the same actions (quasi) simultaneously. Thus, whether interbrain coupling at low frequencies is just a reflection of behavioral coordination or can also stand as a marker of the intentionality behind the cooperative and competitive interactions remains poorly understood.

Alternatively, it is promising to inquire into the role played by high-frequency brain-to-brain synchrony during cooperative and competitive interactions. However, this has been overlooked in some research, either because 20 Hz low-pass filters are used (Lindenberger et al., 2009) or due to problems derived from the implementation of more ecological paradigms that restrict the analysis of the interbrain coupling in a wide frequencies range (Babiloni et al., 2007). The few EEG-based hyperscanning studies that report results in high frequency band show a relationship between the gamma frequency band (>30 Hz) and prosocial interactions. For instance, Kinreich et al. (2017) found an association between brain-to-brain gamma coupling and the degree of social connectedness among interacting partners, while Mu et al. (2017) reported evidence that gamma interbrain coupling is associated with social coordination when humans are exposed to a threat. Additionally, EEG studies in individual participants also suggest an association between gamma synchronization and key components of social cognition such as empathy (Betti et al., 2009) and mentalization (Cohen et al., 2009). Taken together, the general pattern emerging from these sets of studies highlights the involvement mainly of brain-to-brain theta during inter-subject social coordination and a potential role of gamma coupling in prosocial dynamics.

The purpose of the present study is to disentangle the oscillatory interbrain dynamics related to cooperative and competitive social interactions. To achieve this goal, and considering the above body of evidence, we formulate the following hypotheses: (a) In the case of cooperation, we expect that inter-subject motor coordination and shared prosocial goals between participants will be associated with an increase of theta and gamma interbrain coupling, respectively; (b) In the case of competition, we expect that the execution of quasi-simultaneous motor actions between subjects derived from the intention to defeat the other will only increase interbrain coupling in theta band. To test these hypotheses, we simultaneously recorded EEG activity in female-male pairs engaged in a visual cue-target task, which was performed both in a cooperative and competitive manner. The choice of female-male dyads was motivated by the findings reported in the study by Cheng et al. (2015), who found differential co-activation of the prefrontal cortex during collaborative interactions between mixed-sex and same-sex dyads. Specifically, only mixed-dyads exhibited interbrain coupling during cooperation. To avoid physical stimulus confounds or context differences between conditions, the same sensory stimuli and the same stimuli presentation sequence was used in both conditions, such that the only difference was the intention with which the participants performed the task. As an indicator of inter-brain coupling, we measured the phase synchronization values for each pair of electrodes formed between the two EEG caps.

## Materials and Methods

### Participants

A group of 80 healthy university students (40 male, 40 female, range: 18–28 years, mean: 22.58 years) participated in the study. To perform the experiment, participants were randomly ordered into 40 male-female dyads. Members of pairs were not acquainted with each other before the experiment. Furthermore, the empathy quotient (EQ; Baron-Cohen and Wheelwright, 2004) did not differ significantly between dyad members [mean = 40.96, *t*(78) = −0.034, *p* = 0.973, Cohen’s *d* = 0.006). All participants were right-handed native Spanish speakers, with normal or corrected to normal vision/hearing, with self-reported non-history of neurological/psychiatry disorders or drug abuse, and unaware to the purpose of the experiment. All participants gave written informed consent before being tested. The Ethical Committee of the Psychology School of the Pontifical Catholic University of Chile approved the study.

### Experimental Design

**FIGURE 1:**
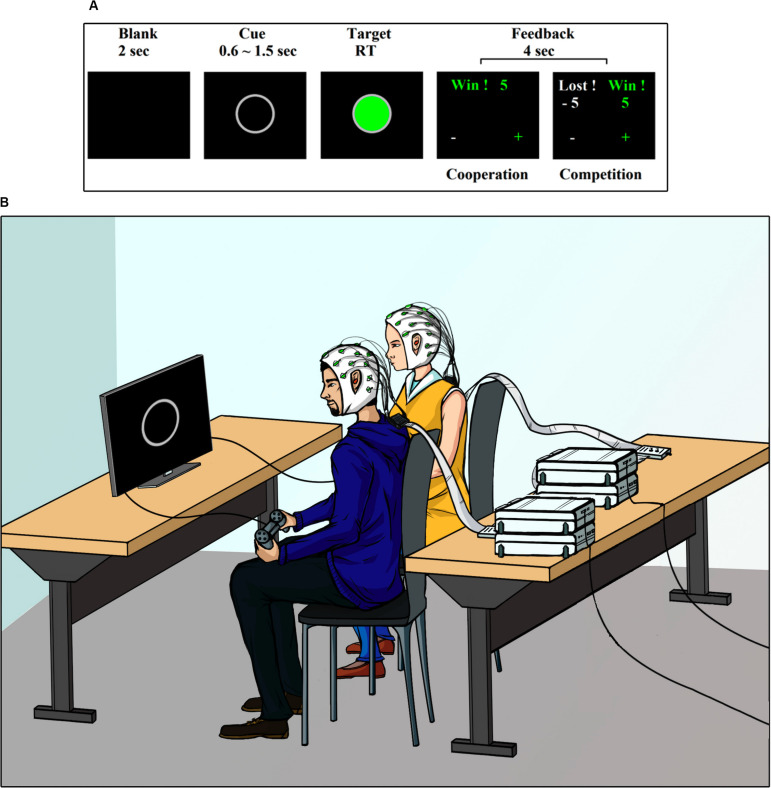
Task and setting. **(A)** Experimental design. **(B)** Experimental setting.

The experiment consisted of a visual cue-target task (Cui et al., 2012; Cheng et al., 2015). Figure 1A shows the sequence of events during the task. Each trial began with the presentation of a 2-s blank, followed by the appearance of a gray ring in the center of the screen. This gray ring was the cue stimulus and had a random duration between 0.6 and 1.5 s. After this random period, the target stimulus was presented. The target stimulus was a green circle with a gray border. Dyad members must press a button on their respective gamepad when viewing the target stimulus. The target stimulus remained on the screen until both subjects responded. After the participants gave their responses, a feedback screen was presented for 4 s.

The same task was carried out by the participants in two different ways, cooperating or competing with each other, as described below.

#### Cooperative Condition

Participants were instructed to work together to earn points as a couple. The dyads earned points if both pressed the button inside a narrow time window. This window was computed as 1/4 of the average dyad reaction time of the preceding trial. Cui et al. (2012) established this value so that the task achieved a reasonable level of difficulty and was sufficiently challenging for subjects to get involved in the task. This adaptive time window allowed for a fixed difficulty of the task while compensating for increasing skill but also increasing fatigue of the subjects as the task progresses. The threshold was calculated as follows:

$$\text{Trial}t\mathrm{is}a\mathrm{winning}\mathrm{trial}\mathrm{if}:\mathrm{abs}\left({\text{RT}}_{\mathrm{S1},t}-{\text{RT}}_{\mathrm{S2},t}\right)<1/4\text{mean}$$

$$\left({\text{RT}}_{\mathrm{S1},t-1},{\text{RT}}_{\mathrm{S2},t-1}\right)$$

where abs() stands for absolute value and RT_*Si,t*_ stands for reaction time for subject *i* during trial *t*.

Finally, a feedback screen indicated if they won or lost points as a couple and their accumulated scores. Moreover, the feedback screen indicated who was faster or slower to respond (plus and minus signs, respectively).

#### Competitive Condition

Participants were asked to respond as quickly as possible, with the intention of defeating their partner. The winner added points to his personal account, while the loser subtracted points. The feedback screen indicated who among the two participants won or lost and the accumulated scores of each of them.

#### Control Condition

To control for the effect of the closeness of motor action between participants as alternative explanation for brain-to-brain coupling, we included a condition in which one of the participants performed the task (active role) while the other only looked at the screen (passive role). Both subjects went through an active and passive role.

Note that for every experimental condition, the physical stimuli always remained the same. Each condition contained 60 trials separated in two blocks of 30 trials each. A 30 s rest period separated both blocks. The order of presentation of the conditions was counterbalanced across dyads.

### Experimental Settings

Figure 1B shows a depiction of the experimental setup. The experiment was conducted in an electromagnetically shielded, soundproof, dimly lit room. The participants were seated side-by-side approximately 50 cm apart. They were both facing a monitor screen Dell Ultra Sharp 1708FP-BLK located at 63 (±3) cm. Participants were asked to use earplugs during the experiment, to avoid that any auditory cue external to the task, could influence its performance. The visual stimuli presentations were controlled with software PsychoPy v1.8. The behavioral responses were recorded with a gamepad. Detailed instructions were provided at the beginning the experiment. The participants were asked to look only at the center of the screen, with their hands holding the gamepad under the table. They were informed that once the study was completed, the earned points would be converted into an additional monetary reward (∼ USD 7 per participant).

### Dual-EEG Data Acquisition

Following the procedures described in Barraza et al. (2019) for the implementation of the EEG hyperscanning setup, the EEG signals of the two participants were simultaneously recorded with two 32-channel BrainAmp DC amplifiers with an actiCap system (Brain Products GmbH). Each subject was connected through an individual Electrode Input Box to one amplifier, which allows for individual Reference and Ground electrodes. Each BrainAmp DC amplifier was battery-powered and coupled using a USB interface (BUA). Signals from the two participants were synchronously recorded in a 64-channel workspace of the BrainVision Recorder software. The data were continuously acquired during the whole experiment. The electrodes were placed at the standard positions based on the extended international 10–20 system. Ground electrodes were placed at AFz location and online reference at channel FCz. Electrode impedances were kept below 10 kΩ. The EEG was filtered online from 0.01 to 100 Hz with a sampling rate of 1000 Hz.

### Dual-EEG Data Preprocessing

The software BrainVision Analyzer v2.0 (Brain Products, GmbH) was used for offline data preprocessing. First, the continuous EEG signal was filtered between 1 and 45 Hz with a zero phase shift Butterworth IIR filter. Then a semi-automatic raw data inspection was applied using a built-in algorithm of the BrainVision Analyzer. This algorithm excluded intervals of 200 ms if there was either an activity of less than 0.5 μV for time windows of 100 ms, voltage steps exceeding 50 μV/ms, or difference of values in intervals exceeding 200 μV/ms. Afterward, Infomax Independent Component algorithm (Lee et al., 1999) was applied. Independent components reflecting blinks and saccades were rejected. Artifact-free trials were re-referenced to average reference in order to minimize artifact sources of synchronization (Bertrand et al., 1985; Nunez et al., 1997).

### Inter-Brain Phase Synchronization Analysis

The EEG signal was segmented into a series of epochs lasting 4000 ms, including 2500 ms preceding the onset to the target stimulus. The segmented EEG signal was analyzed with a sliding-window Fast Fourier Transform (window length, 256 ms; step, 10 ms). For every time window and frequency bin, we obtained the signal phase for frequencies between 1 and 45 Hz, with 1 Hz frequency resolution. The phase component was used to compute the phase-locking value (PLV) as an index of neural synchrony (Lachaux et al., 1999). The PLV reflects the inter-trial variance of the phase difference between participants (Burgess, 2013). In brief, the method involves computing the phase difference inside a time window between all electrode pairs and then assessing the stability of such phase differences through all the trials in the following manner: let ϕ*_*i*_* and ϕ*_*j*_* be unitary vectors representing the phase of signals in electrodes *i* and *j* during time window *t* and frequency bin *f*. The phase difference between such electrodes is given by

ϕij=ϕi⋅ϕj×(ϕj×beingthecomplexconjugateofϕj)

and the PLV is

$$\text{PLV}ij=\text{abs}\left(1/N\times \sum \phi_{ij}\right)$$

with the sum operating through all the trials and *N* being the number of trials. The interbrain phase synchrony index (IBPS) was obtained calculating the PLV for each pair of electrodes (*i*, *j*) between two EEG caps (electrode *i* belonging to cap 1 and electrode *j* to cap 2).

The PLV ranges from 0 to 1, with a value of 1 indicating perfect synchronization (i.e., the phase difference that is perfectly constant throughout the trials), and a value of 0 representing the total absence of synchrony (i.e., phase differences are random). Phase synchronization across an entire trial for each frequency bin was normalized to a 400 ms baseline (from −1372 ms to −972 ms) preceding target stimulus onset. The normalized signal (SN) was obtained by subtracting the average activity of the baseline (μ) from the filtered signal (S) divided by the standard deviation of the baseline (σ), in a frequency-by-frequency manner as in the following equation:

SN=(S-μ)/s

### Surrogate Dataset

To rule out that the IBPS was due to a random phase coincidence, we compared it with IBPS computed in shuffled dyads. Shuffling was performed by randomly forming dyads with participants who did not complete the task together. With this method, we obtained 780 shuffled dyads, which did not include the original dyads. Then, IBPS for each shuffled dyad was computed, as explained before. Thus, we obtained an index of the brain-to-brain synchrony level that would be expected by chance.

### Statistical Analysis

### Behavioral Data

For each version of the task, we calculated the overall average response time (RT) and the RT per block. To measure the closeness of motor response between dyad members, we calculated the difference between the RT of participants 1 and 2.

$$\Delta \text{RT}=\text{abs}\left({\text{RT}}_{\mathrm{S1}}-{\text{RT}}_{\mathrm{S2}}\right)$$

To measure the success of the dyads when cooperating, we calculated the overall percentage of winning trials (PWT) and the PWT per block. The variables were checked for normality via Kolmogorov–Smirnov tests. Variables showing non-parametric distribution were analyzed via Wilcoxon Signed Rank tests or Mann–Whitney U tests as appropriate, otherwise the paired or independent two-tail *t*-test was used. The effect size was calculated with an *r* ratio (non-parametric) or Cohen’s *d* (parametric). Values of 0.2 indicated a small effect, 0.5 a medium effect, and 0.8 or greater a large effect (Cohen, 1988). The α level was set at 0.05 for all tests. Finally, to examine the relationship between interbrain coupling and behavioral performance, a Spearman bivariate correlation analysis was performed. The α level was set at 0.05 for all tests.

#### EEG Data

First, the IBPS matrices were averaged through the trials and electrode pairs, resulting in a grand average time-frequency matrix per dyad. Then, those matrixes were grouped by task type and analyzed by means of paired *t*-tests (*p* < 0.05) in order to identify time-frequency effects more precisely. False discovery rate (*q* < 0.05) was used to correct for multiple comparisons in each of the entry matrix of *p*-values (Benjamini and Yekutieli, 2001). Finally, the selected time-frequency windows were analyzed with repeated measures ANOVA, with the experimental condition (cooperation, competition, individual) as the within-subjects factor and time-frequency windows as dependent variables. Estimates of effect size for ANOVAs were determined with partial η*^2^*. The α level was set at 0.05 for all tests. A Greenhouse–Geisser correction was applied when needed.

For the topographical representation of IBPS, we controlled the probability of false positives resulting from multiple comparisons by choosing a very conservative significance threshold. The threshold was set based on the PLV distribution during the baseline which, being unrelated to the task, was considered to be random. Then, we used this distribution to compute the probability of a given PLV value. Only values exceeding 7 SD of the random distribution were considered to be significant with a *p* < 1 × 10^–12^. By choosing this significance level, one line per analysis window could be explained by chance (for a similar method, see Rodriguez et al., 1999; Uhlhaas et al., 2006; Melloni et al., 2007; Barraza et al., 2014).

## Results

### Behavioral Data

#### Reaction Times

**FIGURE 2:**
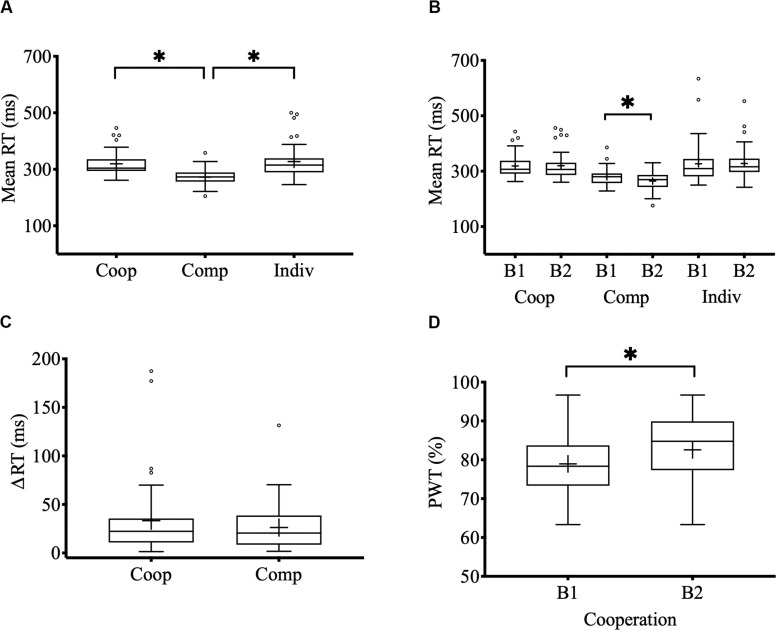
Behavioral performance. **(A)** Mean RT during cooperative, competitive, and individual control tasks. **(B)** Mean RT by blocks and tasks (B1: 1st block; B2: 2nd block). **(C)** Mean RT difference between the members of each dyad in the cooperation and competition task. **(D)** Percentage of winning trials (PWT) in the 1st and 2nd block of the cooperation task. Center lines show the medians; box limits indicate the 25th and 75th percentiles; whiskers extend 1.5 times the interquartile range from the 25th and 75th percentiles; outliers are represented by dots; crosses represent sample means; bars indicate 95% confidence intervals of the means; asterisks show significant differences (*p* < 0.05).

The overall average reaction time of dyads during cooperative interactions was 319.38 ms (SE = 6.88) while competing was 272.10 ms (SE = 4.88). During the individual control task, the reaction time of the dyads was 327.36 ms (SE = 9.62). A Wilcoxon signed-rank test showed that the dyads respond significantly faster when they compete than when they cooperate with each other (*Z* = 5.296, *p* < 0.0001, *r* = 0.837) and faster than the individual task (*Z* = 5.256, *p* < 0.0001, *r* = 0.831) (Figure 2A). Statistical differences between the overall RT during cooperative interactions and the individual control task were not found. Regarding average RTs per block, we found that during competitive interactions the dyads respond significantly faster in the 2nd block (264.41 ms, SE = 5.50) than in the 1st block (279.76 ms, SE = 4.96) (*Z* = −3.495, *p* < 0.0001, *r* = 0.552) (Figure 2B). Statistical differences between blocks during cooperative interactions and individual tasks were not found. Finally, regarding the closeness of RT between dyads members, we found that the ΔRT (difference in RT between participants 1 and 2) during cooperative interactions was 33.20 ms (SE = 6.36) while competing was 26.20 ms (SE = 3.83) (Figure 2C). There were no statistical differences between ΔRT of cooperative and competitive interactions.

#### Percentage of Winning Trials

The overall PWT during cooperative interactions was 80.75 % (SE = 1.22). A Wilcoxon one sample signed-rank test showed that the PWT was significantly greater than chance (*Z* = 5.514, *p* < 0.0001, *r* = 0.871). Regarding the PWT per block, we found that success in cooperation improved significantly in the 2nd block (82.57%, SE = 1.35) compared with the 1st block (78.96%, SE = 1.33) (*Z* = −2.869, *p* = 0.004, *r* = 0.453) (Figure 2D).

### Interbrain Phase Synchrony

#### Gamma Interbrain Coupling

**FIGURE 3:**
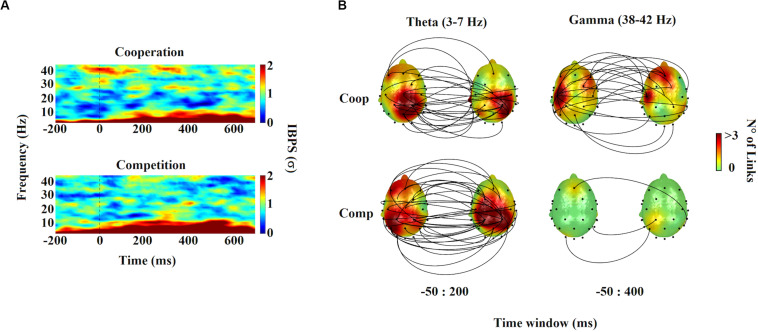
Time-frequency charts and interbrain distribution. **(A)** Time-frequency maps of the interbrain phase synchrony (IBPS) for cooperation and competition task. Frequency and time are indicated in the *y*- and *x*-axis of the maps, respectively. The color bar shows the magnitude of the IBPS in standard deviations (σ). Time 0 indicates the onset of the target stimulus. **(B)** Topographic distribution of the theta and gamma IBPS. Lines connect pairs of electrodes displaying significant brain synchronization between members of dyads (*p* < 0.0001). The color over scalps indicates the number of links per electrode.

The results are presented in Figure 3A. We found transient brain-to-brain gamma activity (38–42 Hz) during cooperative interactions around and after the onset of the target stimulus (win1: −50–100 ms; win2: 300–400 ms). Repeated-measures ANOVA revealed that gamma interbrain coupling during the cooperative task was significantly higher compared with the competitive (win1: *F*_1,39_ = 12.407, *P* = 0.001, η^2^*p* = 0.252; win2: *F*_1,39_ = 4.618, *P* = 0.038, η^2^*p* = 0.106) and individual control tasks (win1: *F*_1,39_ = 7.254, *P* = 0.01, η^2^*p* = 0.157; win2: *F*_1,39_ = 5.167, *P* = 0.029, η^2^*p* = 0.117). Statistical differences between competition and individual control tasks were not found. Regarding the control of spurious interbrain synchrony effects, we found that the average of shuffled gamma IBPS varied little over the entire observation window compared to gamma IBPS induced by cooperative interactions. In the case of competitive interaction and the individual control task, gamma IBPS did not differ mostly from background random gamma fluctuations. Finally, the topographic representation of the gamma interbrain network showed a dense pattern of connectivity during cooperative interactions, distributed mainly between left frontal-temporal electrodes (Figure 3B).

#### Theta Interbrain Coupling

The results are presented in Figure 3A. We found a strong and sustained increase in brain-to-brain theta coupling (3–7 Hz) during both cooperative and competitive interactions, which was slightly higher for competition than cooperation, from -50 ms prior to 200 ms after onset target stimulus (F_1,39_ = 5.212, *P* = 0.028, η^2^*p* = 0.118). Theta brain-to-brain coupling during the individual control task was not found. Regarding the control of spurious interbrain synchrony effects, the average of shuffled theta IBPS varied little over the entire observation window compared with the theta IBPS induced by cooperative and competitive interactions. The theta IBPS in individual control task did not differ from background random theta fluctuations. Concerning the topographic representation of theta interbrain network, we found that both cooperation and competition elicited the emergence of similar brain-to-brain connectivity patterns, distributed mainly between central frontal-parietal electrodes, being denser during competitive interactions (Figure 3B).

#### Correlation Between Interbrain Coupling and Behavioral Performance

**FIGURE 4:**
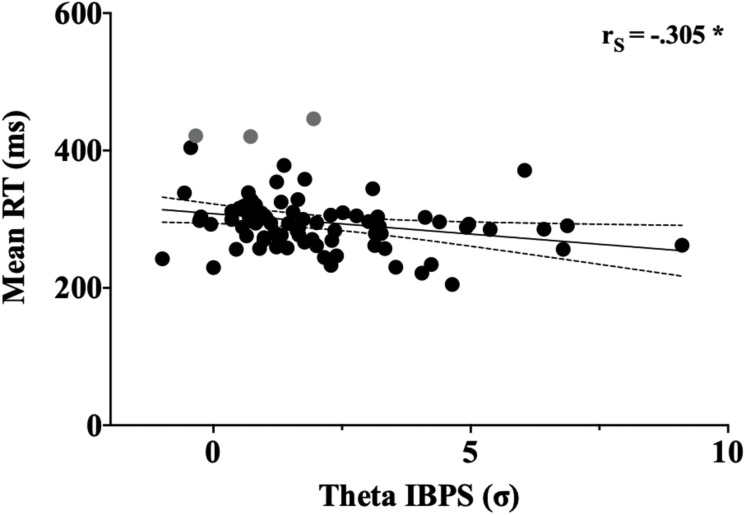
Correlation between theta IBPS values and reaction time. Each dot represents a single dyad. Time and theta IBPS are respectively indicated in the *y*- and *x*-axes of the graph, respectively. Asterisk indicates significant correlation (*p* < 0.05). Bivariate outliers excluded from the robust correlation are shown in gray.

To evaluate the degree of association between interbrain coupling and motor response, a Spearman bivariate correlation analysis was computed between the IBPS values and the mean reaction times of the dyads (Figure 4). The analysis revealed a significant negative association between interbrain theta coupling and reaction times [Rs (80) = −0.305, *p* = 0.0059), meaning that stronger theta synchronization correlated with shorter reaction times. Correlation between interbrain gamma coupling and reaction times were not found. To test whether bivariate outliers affected the association between interbrain theta coupling and reaction times, we performed a skipped correlation (Pernet et al., 2013). This method excludes bivariate outliers and provides a more robust estimate of the relationship between variables. The robust correlation analysis confirmed the presence of a negative and significant association between interbrain theta synchronization and reaction times [skipped Rs = −0.279, 95% CI = (−0.494 to −0.043)].

## Discussion

In the present study, we analyzed the behavioral performance and oscillatory interbrain dynamics of dyads involved in cooperative and competitive interactions. The experimental design allowed us to assess differences in the cooperative and competitive mental stance (Cho et al., 2020) during an otherwise similar joint action. Our findings add evidence to support the idea of idiosyncratic neurodynamics of interbrain coupling for cooperative and competitive social interactions. In line with previous studies (Balconi and Vanutelli, 2017b; Liu et al., 2018) our results show an increased theta PLV brain-to-brain coupling associated with the social interaction (i.e., both competition and cooperation). Furthermore, theta coupling was associated with faster reaction times. We also observed increased activity in gamma band frequency range (38–42 Hz) only for cooperative interactions. Taken together the results suggest that the enhanced theta inter-brain is a marker of inter-subject motor action and that gamma brain-to-brain coupling could be the neural signature of cognitive processes underlying cooperation, such as shared intentionality (Tomasello and Carpenter, 2007).

In behavioral terms, our results revealed that cooperative interactions involved an efficient behavioral co-regulation among dyads’ members, with a steady improvement of this behavior through trials. Specifically, we observed an enhancement of the PWT in the second block of the cooperation task, which suggests a kind of “behavioral tuning” between cooperative partners across time, in pursuit of the fulfillment of a shared goal. The above findings are in line with proposals stating that the behavioral co-regulation during prosocial interactions is a foundation for effective social exchange (Semin and Cacioppo, 2008; Miles et al., 2009). As expected, competition reduced the reaction times of the dyads’ members. Considering that our task did not include “no-go” or distracting stimuli that must be kept under control, it is likely that to meet the goal of defeating the opponent, the action of inhibitory control mechanisms is reduced (Kenner et al., 2010), incurring a more impulsive motor response to improve the reaction times but at the cost of accuracy (Delorme et al., 2007; Bogacz et al., 2010). This interpretation is consistent with studies supporting the relationship between reaction time and efficiency of decision-making (Forstmann et al., 2008, 2010). Another aspect to consider is the quasi-simultaneity of movement (button press) between participants, both cooperating and competing. Comparison of the ΔRT of the pairs did not show any significant difference between cooperative and competitive conditions. This indicates that the closeness of the motor response between participants does not seem to facilitate understanding of brain-to-brain coupling, which is in agreement with previous studies (Cui et al., 2012; Cheng et al., 2015).

### Theta Interbrain Coupling and Motor Coordination During Social Interactions

Our results showed a strong brain-to-brain coupling oscillating at low frequency during cooperative and competitive interactions, being even stronger when dyads’ members competed with each other. Interestingly, these differences in the theta interbrain level were associated with the behavioral performance of the dyads. Specifically, we found that as the reaction times of the dyads decreased, the theta brain-to-brain coupling increased. Regarding the individual control task, our findings revealed that if one dyad member responded to the task while the other only watched the screen, then theta interbrain coupling was not elicited. These findings led us to think that theta interbrain coupling could be an index of the inter-subject motor coordination, regardless of whether such behavioral interaction were intentional. This interpretation is in line with previous EEG studies, where simple imitation and spontaneous coordination of movements between participants also induced interbrain coupling at low frequencies (Tognoli et al., 2007; Dumas et al., 2010). Alternatively, it is possible to hypothesize that theta interbrain coupling could be representing the action of common cognitive functions during cooperative and competitive interactions. For instance, Decety et al. (2004), in an fMRI study, reported that both types of social behavior activate a frontal-parietal neural network supporting executive processes. In terms of EEG dynamics, numerous reports associate theta activity with executive functions such as working memory (Fell and Axmacher, 2011; Clouter et al., 2017) and cognitive control mechanisms (Cavanagh and Frank, 2014; Cooper et al., 2019). From this perspective, the increase in theta brain-to-brain coupling could be considered an index of the participation of executive processes during cooperative and competitive interactions, either to co-regulate the behavior between subjects or to improve individual behavioral performance without considering the behavior of the other.

### Gamma Interbrain Coupling as a Marker of Prosocial Behavior

Our results showed the emergence of a transient brain-to-brain network oscillating in gamma frequency band during cooperative interactions. It is striking that this interbrain gamma coupling was not observed when the members of the dyad competed with each other or when one member of the dyad pressed the button while the other just watched the screen. Additionally, we found that this increase in interbrain gamma coupling was not associated with the reaction time of dyads, and it was much larger than expected by simple random phase coincidence. To elucidate the potential role of gamma interbrain coupling during social interactions, let us recall that cooperative and competitive tasks were performed under the same conditions of stimulation and evoked similar inter-subject motor coordination, such that the only difference between conditions was the intention with which participants performed the tasks (collaborate to achieve a shared goal or not). In electrophysiological terms, recent EEG studies have associated oscillatory gamma activity with social cognitive skills, such as empathy (Betti et al., 2009), mentalization (Cohen et al., 2009), social bonding (Kinreich et al., 2017), and prosocial behavior (Mu et al., 2017). Taken together, these antecedents support the idea that gamma brain-to-brain coupling could be an electrophysiological marker of shared intentionality (Tuomela, 2006; Tomasello and Carpenter, 2007; Gallotti and Frith, 2013). Shared intentionality involves meshing and sharing of psychological states with others (Call, 2009), with the objective of co-creating joint intentions and joint commitments in cooperative endeavors (Tomasello, 2009). According to the fMRI study conducted by Wittmann et al. (2016), the ability to mesh our own intentions with the intentions of others depends on the action of a neural circuit located in the dorsomedial frontal region (area 9). The action of this brain region would facilitate inter-subject cognitive coupling and the pursuit of shared goals during cooperative interactions. To combine our own intentions with the intentions of the other, a type of predictive coding seems necessary, which allows us predict the other’s future mental state, compare it with our own mental state, and thus adjust our social behavior in the present. Interestingly, this neural mechanism has recently been reported by Thornton et al. (2019), who found that the adjustment of current social behavior based on the prediction of the other’s future mental state, depended on the action of the medial prefrontal cortex. Similar findings have been found in pairs of monkeys that perform cooperative tasks (Ferrari-Toniolo et al., 2019). Specifically, a unique set of dorsal premotor neurons called “joint-action cells” was found to discharge preferentially during cooperative tasks involving interactive visuomotor coordination between co-acting monkeys. Regarding the role of the brain-to-brain gamma coupling in the emergence of shared intentionality during cooperative interactions, we propose that the recurrent action of integrating and sharing psychological states with the other would reduce the randomness of gamma phases in the dyads’ members, thus increasing the probability of interbrain coupling at high frequencies. Specifically, we suggest that when two (or more) self-organized systems enter into recurrent cooperative interactions, each system triggers emerging local dynamics for the regulation of social behavior. This initial stage would be characterized by high randomness of gamma phases in both systems. Subsequently, reciprocity in cooperative behavior would act as a social reward (Rilling et al., 2002; Decety et al., 2004; Hare et al., 2010), which operates as an order parameter constraining the possible gamma phase states between subjects, thus facilitating brain-to-brain coupling in the gamma range.

It should also be noted that this study has some limitations. First, the interbrain synchronization was assessed by measuring the consistency of the phase difference across trials. This is fundamentally different from the measurement of the phase covariance across time inside the trial (Burgess, 2013). Thus, it could be tentatively argued that the results herein are more influenced by systematic differences between conditions. However, we consider that we ruled out this possibility with the applied statistical analysis. Each condition was compared against a condition-specific surrogate data set (i.e., created for each condition). Thus surrogate data would contain all possible systematic bias, preventing the emergence of statistical effects. Second, additional control conditions would be desirable. For instance, a condition in which the participants are jointly interacting with the computer would allow the removal of synchronization with a human agent (Astolfi et al., 2020). With this type of control, we would be able to further interpret the results in terms of agency effects. Future studies should adopt improved experimental control (Astolfi et al., 2020) and the use of data-association measures addressing covariance between brain signals with precise localization of synchrony in time and frequency (e.g., Hemakom et al., 2017). Additionally, the use of source reconstruction techniques will be highly desirable for more precise localization of the underlying areas supporting the differential processing.

## Conclusion

In summary, the present study contributes to previous EEG hyperscanning research on the neurodynamic of social interactions by showing that the inter-subject motor coordination and prosocial intentions can be distinguished according to the frequency of oscillation in which the brains are coupled. Specifically, our findings revealed that processes common to cooperative and competitive social interactions, such as executive control of movements, are best represented by the interbrain coupling in the theta band, while the cognitive aspects that characterize cooperative interactions, such as shared intentionality, are expressed in the brain-to-brain coupling in the gamma band.

## Data Availability Statement

The datasets generated for this study are available on request to the corresponding author.

## Ethics Statement

The studies involving human participants were reviewed and approved by the Ethical Committee of the Psychology School of the Pontifical Catholic University of Chile. The patients/participants provided their written informed consent to participate in this study.

## Author Contributions

PB and ER conceived the research, designed the study, and performed the EEG data acquisition. All authors performed data analysis and interpreted findings, drafted the manuscript, and approved the final draft for submission.

## Conflict of Interest

The authors declare that the research was conducted in the absence of any commercial or financial relationships that could be construed as a potential conflict of interest.
